# Salivary and GCF Vitamin C Levels: Implications for Local and Systemic Periodontal Defense

**DOI:** 10.1155/ijod/8367922

**Published:** 2025-12-03

**Authors:** Nada Tawfig Hashim, Muhammed Mustahsen Rahman, Md Sofiqul Islam, Vivek Padmanabhan, Nallan C. S. K. Chaitanya, Riham Mohammed, Pooja Shivappa, Nancy Soliman Farghal, Hassan Khalifa Nayef, Manar Salah Talib, Sadiah Fathima, Nurain Mohammad Hisham, Aya Chaik

**Affiliations:** ^1^Periodontics Department, RAK College of Dental Sciences, RAK Medical and Health Sciences University, Ras Al Khaimah, UAE; ^2^Operative Department, RAK College of Dental Sciences, RAK Medical and Health Sciences University, Ras Al Khaimah, UAE; ^3^Department of Pediatric and Preventive Dentistry, RAK College of Dental Sciences, RAK Medical and Health Sciences University, Ras Al Khaimah, UAE; ^4^Oral Medicine and Radiology Department, RAK College of Dental Sciences, RAK Medical and Health Sciences University, Ras Al Khaimah, UAE; ^5^Oral Surgery Department, RAK College of Dental Sciences, RAK Medical and Health Sciences University, Ras Al Khaimah, UAE; ^6^Translational Medical Research Centre, RAK Medical and Health Science University, Ras Al Khaimah, UAE; ^7^Manipal School of Life Sciences, Manipal Academy of Higher Education, Manipal, India; ^8^Department of Endodontics, RAK College of Dental Sciences, RAK Medical and Health Sciences University, Ras Al Khaimah, UAE; ^9^Department of Dental Biomaterials, Faculty of Dentistry, Tanta University, Tanta, Egypt

**Keywords:** antioxidant, gingival crevicular fluid, grading, oxidative stress, periodontitis, saliva, staging, vitamin C

## Abstract

**Background:**

Periodontitis is a chronic inflammatory condition influenced by oxidative stress, which contributes to tissue destruction. Vitamin C, a key antioxidant, is essential for maintaining periodontal health through its role in collagen synthesis and free radical scavenging.

**Objective:**

This study aimed to assess vitamin C levels in saliva and gingival crevicular fluid (GCF) across different stages of periodontitis and explore their potential as biomarkers of disease progression.

**Methods:**

A cross-sectional study was conducted involving 43 participants categorized into healthy, mild, moderate, and severe periodontitis groups according to the 2017 classification. Saliva and GCF samples were collected and analyzed for vitamin C using validated biochemical methods. Statistical analysis included Kruskal–Wallis tests and pairwise comparisons.

**Results:**

Salivary vitamin C levels were significantly higher in healthy individuals and progressively decreased with increasing disease severity. GCF vitamin C concentrations were consistently higher than in saliva across all groups, with elevated levels in both healthy and severe periodontitis participants. However, intergroup differences in GCF levels were not statistically significant.

**Conclusion:**

The study reveals distinct profiles of vitamin C in saliva and GCF, highlighting its local and systemic antioxidant roles. These findings suggest that vitamin C may serve as a useful biomarker in periodontal disease assessment.

## 1. Background

Periodontitis, a chronic inflammatory disease that compromises the supporting structures of teeth, remains a significant public health burden due to its high prevalence and strong association with systemic health complications. If untreated, periodontitis leads to progressive destruction of the periodontal ligament and alveolar bone, culminating in tooth mobility and eventual tooth loss [1, 2]. The American Academy of Periodontology (AAP) and the European Federation of Periodontology (EFP) have jointly introduced a multidimensional staging and grading system for diagnosing periodontitis. While staging reflects disease severity and complexity of treatment, grading assesses the biological risk of progression and systemic implications. This framework categorizes periodontitis into four stages (I–IV) and three grades (A–C), enabling a more comprehensive understanding of the disease [3].

The pathogenesis of periodontitis is multifactorial, involving intricate interactions between pathogenic bacteria and the host immune response, with oxidative stress playing a central role. Oxidative stress, defined as an imbalance between the production of reactive oxygen species (ROS) and the antioxidant defense system, drives tissue destruction and exacerbates inflammation in periodontal disease [4].

Ascorbate (vitamin C), a potent water-soluble antioxidant, is integral to the host's defense system. It enhances immune response, supports collagen synthesis, and is vital for periodontal tissue repair and regeneration [5–7]. Given its critical role in mitigating oxidative stress and promoting periodontal health, ascorbate has garnered interest as a potential biomarker for periodontal disease severity. Reduced levels of vitamin C in the body can reflect heightened oxidative stress, impaired antioxidant defenses, and an exacerbated inflammatory response, all of which are hallmarks of periodontitis [8, 9].

Despite evidence that individuals with periodontitis exhibit lower plasma antioxidant levels than healthy controls [10–12], limited research has specifically investigated ascorbate levels in saliva and gingival crevicular fluid (GCF) across different stages of periodontitis. By quantifying vitamin C levels in these fluids, this study seeks to elucidate the oxidative stress profile associated with periodontal disease progression and to identify potential therapeutic targets.

The findings may have significant implications for periodontal therapy. Insights into ascorbate dynamics could pave the way for innovative antioxidant-based treatment strategies, including dietary supplementation or pharmacological formulations, aimed at enhancing antioxidant defenses. Such interventions might not only control periodontal destruction but also reduce the systemic health risks associated with periodontitis, such as cardiovascular disease and diabetes [13, 14]. The dual measurement of vitamin C in saliva and GCF provides a comprehensive perspective on its role in periodontal health and disease. While saliva reflects systemic antioxidant status, GCF offers a localized snapshot of oxidative stress and tissue health at the site of periodontal inflammation. Investigating the interplay between these fluids could deepen our understanding of how systemic and local factors converge in periodontal pathogenesis [15, 16].

Therefore, this study explores vitamin C levels in both saliva and GCF, two distinct yet interconnected oral environments. Saliva, being easily accessible, provides a systemic overview of antioxidant status, while GCF represents a more localized biochemical milieu, directly reflecting periodontal tissue conditions. Measuring vitamin C levels in these fluids offers a unique opportunity to understand the systemic and local dynamics of oxidative stress and antioxidant defense in periodontitis.

## 2. Methodology

This cross-sectional study assessed vitamin C levels in saliva and GCF among individuals with varying stages of periodontitis. Participants were grouped into four categories: healthy controls, mild periodontitis, moderate periodontitis, and severe periodontitis. The study was conducted at RAK College of Dental Sciences (RAKCODS) over 6 months, from October 2023 to April 2024. The research was approved by the RAK Medical and Health Sciences University Research Ethics Committee under approval number RAKMHSU-REC-043-2022/23-UG-D.

The sample size was set at 43 participants due to practical considerations, with a statistical power of 0.36 calculated using G^*⁣*^*∗*^^Power software, assuming a medium effect size (Cohen's *f* = 0.25) and an alpha level of 0.05. Participants were allocated proportionally across the groups, ensuring representation from all stages of periodontitis and healthy controls.

### 2.1. Participants and Grouping

Participants were recruited through convenience sampling from the outpatient department at RAKCODS between October 2023 and April 2024. All participants underwent a comprehensive periodontal examination, and diagnoses were made based on the 2017 AAP/EFP classification system, which incorporates staging (I–IV) and grading (A–C) of periodontitis [3].

For the purposes of statistical analysis, participants were subsequently grouped into broader clinical severity categories: healthy, mild, moderate, or severe periodontitis, derived from the original staging and grading diagnoses [2] (Table 1).

Inclusion criteria is as follows:

• Adults aged 18–55 years.
• Provided written informed consent.
• Diagnosed with periodontitis (any stage or grade) or confirmed healthy periodontium.

Exclusion criteria is as follows:

• Current or recent (<6 months) use of vitamin C supplementation or antioxidant therapy.
• History of periodontal therapy within the last 6 months.
• Systemic diseases or medications affecting periodontal health.
• Pregnancy or lactation.
• Current smokers or alcohol users.
• Participants unable to complete or continue the study for any reason (e.g., withdrawal, noncompliance).

### 2.2. Clinical Examination

Participants underwent a comprehensive periodontal examination to measure clinical attachment loss (CAL) and probing depth (PD) using a University of Michigan probe with William markings. The assessment included radiographic evaluation to confirm the extent of alveolar bone loss, which was critical for accurate staging and grading. Orthopantomograms (OPGs) were obtained for all participants to ensure consistency in radiographic evaluation. Sites with the highest CAL and the most severe clinical signs of inflammation were identified for GCF collection. All periodontal diagnoses were independently verified by two calibrated examiners. Interexaminer reliability was assessed using Cohen's kappa statistic, demonstrating substantial agreement (kappa = 0.8), ensuring diagnostic consistency.

### 2.3. Sample Collection

Saliva and GCF were collected from all participants under standardized conditions:

• Saliva collection: Unstimulated saliva samples were collected between 9 AM and 11 AM to minimize diurnal variation in antioxidant levels. Participants refrained from eating, drinking, or performing oral hygiene procedures for at least 1 h before collection. Saliva was collected by the spitting method over 5 s into sterile collection tubes, which were immediately stored at −80°C.
• GCF collection: GCF was obtained from the site with the most severe periodontal involvement in periodontitis patients and a healthy site in control participants. The selected site was isolated with cotton rolls, gently dried, and an absorbent paper strip was inserted into the gingival sulcus for 30 s [2]. Strips were then placed in Eppendorf tubes and stored at −80°C. GCF samples were immediately placed into phosphate buffer (pH 7.4) and gently shaken to release absorbed fluids into the buffer solution. After removing the absorbent paper, the tubes were stored at 4°C until analysis. The centrifuged supernatant was used for vitamin C assays.

### 2.4. Biochemical Analysis

The collected samples were prepared for biochemical analysis as follows:

• Saliva and GCF samples were centrifuged at 3000 rpm for 15 min to remove debris, and the supernatant was collected.
• Vitamin C levels were measured using the 2,4-dinitrophenylhydrazine method, which quantifies dehydroascorbic acid converted to ascorbic acid. The spectrophotometric absorbance was measured at 540 nm using a UV-visible spectrophotometer (JENWAY 6505, Unico).

### 2.5. Statistical Analysis

Data analysis was performed using SPSS software. Descriptive statistics summarized vitamin C concentrations in saliva and GCF across the groups. The Kruskal–Wallis test was applied to assess differences among groups, followed by post hoc pairwise comparisons. A *p*-value of  < 0.05 was considered statistically significant.

## 3. Results

### 3.1. Data Description

The study included 43 participants who were classified based on the severity of their periodontal condition, ranging from healthy individuals to those with varying stages and grades of periodontitis (stages I–IV, grades A–C). Of the participants, 58% were male, and 42% were female, with a mean age of 38.8 ± 8.8 years.

The severity of periodontal disease among the study participants was categorized into mild, moderate, or severe based on CAL and corresponding to the stages and grades of periodontitis. Mild periodontitis was observed in 9 participants, characterized by a CAL of 1–2 mm. Moderate periodontitis included 13 participants, exhibiting a CAL of 3–4 mm, representing an intermediate stage between mild and severe periodontitis. Severe periodontitis, the most advanced form of the disease, was identified in 9 participants with a CAL exceeding 5 mm, indicating extensive periodontal destruction [17]. In contrast, the healthy group comprised 13 participants with intact periodontium, showing no evidence of CAL or alveolar bone loss [18]. (Table 1).

### 3.2. Vitamin C Concentrations in Saliva by Disease Severity

**Figure 1:**
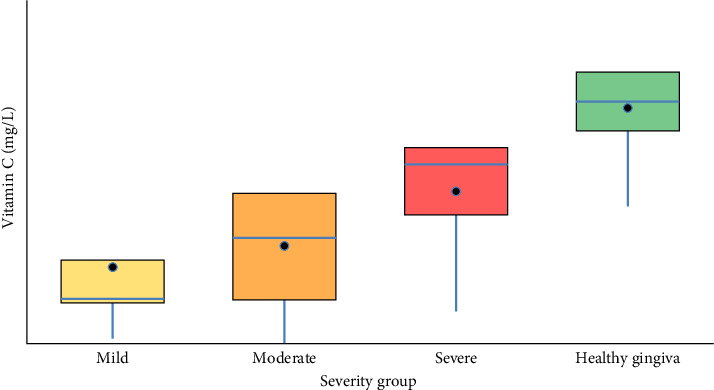
Boxplot showing the distribution of vitamin C levels in saliva across periodontal severity groups (mild, moderate, severe, and healthy gingiva). Boxes represent the interquartile range (IQR), the horizontal line marks the median, whiskers denote minimum and maximum values, and black dots denote group means. Normality of salivary vitamin C distribution was assessed using the Shapiro–Wilk test (*W* = 0.952, *p*=0.069).

The salivary vitamin C levels exhibited significant variation across the periodontal severity groups (Figure 1). The Shapiro–Wilk test and boxplots indicated that the “mild” and “severe” periodontitis groups did not follow a normal distribution (*p*  < 0.05), while the “moderate” and “healthy gingiva” groups were normally distributed. Given the mixed normality results, a Kruskal–Wallis test was performed, revealing statistically significant differences in median vitamin C levels among the groups (Kruskal–Wallis statistic = 20.46, *p*=0.000136). The median vitamin C level was highest in the “healthy gingiva” group (4.7172), followed by the “severe periodontitis” group (3.4907), the “moderate periodontitis” group (2.0600), and the “mild periodontitis” group (0.8743). Pairwise comparisons using Tukey's HSD test indicated significant differences between the “healthy gingiva” group and all other groups (*p*  < 0.05). However, no statistically significant differences were observed among the periodontitis severity groups themselves (mild, moderate, and severe) (Tables 2 and 3).

### 3.3. Vitamin C Concentrations in GCF by Disease Severity

The concentrations of vitamin C in GCF also varied across the severity groups. Patients with healthy gingiva and those with severe periodontitis had the highest median vitamin C levels, both recorded at 4.9625. The “mild periodontitis” group exhibited slightly lower levels, with a median of 4.4719, while the “moderate periodontitis” group demonstrated the lowest median vitamin C level at 0.3270. The Kruskal–Wallis test for these groups yielded a statistic of 7.32 (*p*=0.062), indicating that while differences in GCF vitamin C levels approached statistical significance, they did not meet the conventional threshold of *p*  < 0.05. (Table 4).

### 3.4. Comparison of Vitamin C Levels in Saliva and GCF

**Figure 2:**
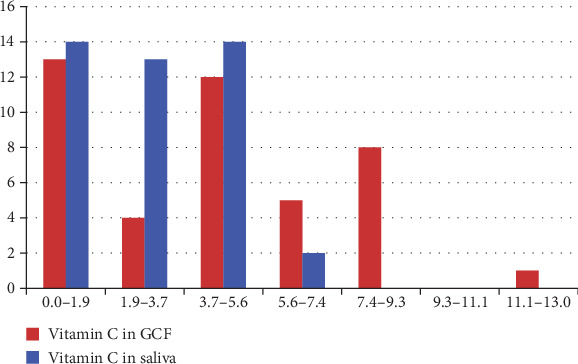
Distribution of vitamin C levels in gingival crevicular fluid (GCF) and saliva. This clustered histogram illustrates the frequency distribution of vitamin C concentrations measured in GCF (red bars) and saliva (blue bars). The *x*-axis represents vitamin C concentration ranges (mg/L), while the *y*-axis shows the frequency of observations within each interval. The figure demonstrates visibly different distribution patterns between the two fluids, with GCF showing a higher proportion of lower concentration values and saliva showing more variability across mid-range concentrations. These non-normal and skewed distribution patterns support the use of nonparametric statistical testing for comparing vitamin C levels between GCF and saliva.

When comparing vitamin C levels in saliva and GCF, the distribution in both fluids was found to be skewed, necessitating a nonparametric approach (Figure 2). The median vitamin C concentration was higher in GCF (4.5537) than in saliva (3.1637). A Wilcoxon signed-rank test confirmed a statistically significant difference between these paired samples (statistic = 284.0, *p*=0.022). This finding suggests that vitamin C levels are consistently higher in GCF compared to saliva, highlighting the potential role of GCF in localized antioxidant defense within the periodontal microenvironment (Table 5).

**Figure 3:**
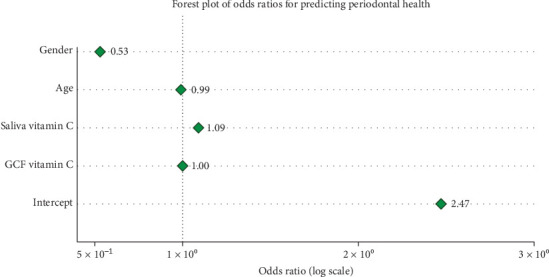
Forest plot of odds ratios for predictors of periodontal health status. The figure displays the odds ratios derived from a binary logistic regression model examining key predictors of periodontal health. The variables included in the model were gender, age, salivary vitamin C concentration, and GCF vitamin C concentration. Odds ratios are plotted on a logarithmic scale, and the vertical dashed line represents the line of no effect at OR = 1.0. Values to the right of this line indicate an increased likelihood of periodontal health, whereas values to the left indicate reduced likelihood. Salivary vitamin C showed the strongest positive association with periodontal health (OR = 1.09), while gender (OR = 0.53) showed a negative association. Each marker represents the point estimate of the odds ratio, and horizontal alignment aids in visual comparison across predictors.

A binary logistic regression was conducted to assess whether vitamin C concentrations in GCF and saliva can independently predict periodontal health status while controlling for age and gender. Although GCF demonstrated significantly higher median vitamin C levels compared to saliva (4.55 vs. 3.16 µg/mL, *p*=0.022), the model again showed perfect separation, indicating nearly flawless classification of health status based on the chosen predictors (Table 6). According to coefficient estimates, salivary vitamin C was positively associated with periodontal health (OR = 1.09), whereas GCF vitamin C had minimal influence (OR ≈ 1.00). Age and male gender were negatively correlated with being healthy. These findings suggest that while GCF contains higher vitamin C concentrations, salivary vitamin C may serve as a more predictive and clinically relevant biomarker for periodontal health (Figure 3).

## 4. Discussion

Periodontal disease is a complex, multifactorial condition influenced by both local and systemic factors, including oxidative stress. This study focused on investigating the role of vitamin C, a key antioxidant, in the context of periodontal health and disease. By evaluating its levels in both saliva and GCF, the study provides a unique biochemical perspective on how local and systemic antioxidant defenses may differ across varying stages of periodontal severity.

### 4.1. Vitamin C Concentrations in Saliva by Disease Severity

The significant differences in salivary vitamin C levels across the periodontal severity groups observed in this study are consistent with existing literature linking oxidative stress to periodontal disease. Healthy individuals exhibited the highest vitamin C levels, reflecting better antioxidant defenses and reduced oxidative stress compared to periodontitis patients. Previous studies have consistently demonstrated lower salivary antioxidant levels, including vitamin C, in individuals with periodontal disease compared to periodontally healthy subjects. For instance, Isola et al. [19] and Diab-Ladki et al. [20] reported significant reductions in salivary vitamin C in periodontitis patients, emphasizing the role of antioxidants in mitigating disease-related oxidative damage. Similarly, Sculley and Langley-Evans [21] highlighted the depletion of vitamin C in saliva as a consequence of increased oxidative stress in the periodontal microenvironment.

However, the lack of significant differences in vitamin C levels among the mild, moderate, and severe periodontitis groups presents an intriguing finding. This may reflect a threshold effect, where vitamin C depletion occurs early in the disease process and plateaus regardless of disease progression. Supporting this idea, Sculley and Langley-Evans [21] proposed that once oxidative stress disrupts vitamin C metabolism, further disease progression may not decrease salivary levels. Other researchers, such as Buzatu et al. [22], have suggested that dietary intake, supplementation, or improved oral hygiene practices in patients with advanced disease might contribute to similar vitamin C levels across varying severities. This raises the question of whether salivary vitamin C can adequately reflect incremental differences in disease severity. Although vitamin C deficiency is widely associated with increased periodontal risk, the elevated salivary vitamin C levels observed in the severe periodontitis group may reflect a compensatory biological response. As the severity of periodontal inflammation increases, the host may mobilize antioxidants like vitamin C from systemic stores to inflamed tissues in an attempt to counteract oxidative damage. This aligns with findings by Ahmadi-Motamayel et al. [15], who reported elevated antioxidant markers in patients with advanced disease, suggesting an adaptive immune response. Additionally, it is possible that patients with more severe disease had modified behaviors, such as improved dietary habits or oral care, following diagnosis, which may have contributed to their higher vitamin C levels. Nonetheless, contrasting evidence from Saraç Atagün et al. [23] suggests that severe disease states might further exhaust antioxidant reserves. These discrepancies highlight the complex relationship between oxidative stress, antioxidant defense, and periodontal disease progression [11, 24].

### 4.2. Vitamin C Concentrations in GCF by Disease Severity

Unlike saliva, the vitamin C concentrations in GCF did not show statistically significant differences among severity groups. This finding is supported by evidence from Nishida et al. [25], who observed that GCF vitamin C levels are influenced not only by disease severity but also by systemic vitamin C intake, gingival inflammation, and local immune responses. The absence of significant differences in this study may reflect a compensatory response in GCF, where local homeostatic mechanisms work to maintain vitamin C levels irrespective of disease severity [26]. The high levels of vitamin C observed in both healthy and severe periodontitis groups suggest a dual role for this antioxidant. In healthy individuals, elevated vitamin C may reflect a robust antioxidant defense supporting tissue integrity. Conversely, in severe periodontitis, elevated GCF vitamin C could indicate an adaptive response to increased oxidative stress and inflammation, as proposed by Green and Ford [27]. The markedly lower levels in moderate periodontitis, however, raise questions about potential disruptions in protective mechanisms during this disease stage. Williams and Singh [28] suggested that intermediate stages of periodontal disease might involve a tipping point where oxidative damage outpaces antioxidant defenses, leading to the observed reduction in vitamin C levels. While the lack of statistical significance in GCF vitamin C levels might suggest a uniform response across severity groups, the *p*-value approaching significance highlights the need for further investigation. Studies with larger sample sizes, controlled vitamin C intake, and more sensitive measurement techniques may clarify whether subtle variations in GCF vitamin C exist. Additionally, longitudinal studies could help determine whether GCF vitamin C levels change over time in response to disease progression or therapeutic interventions.

### 4.3. Comparison of Vitamin C Levels in Saliva and GCF

The significant difference in vitamin C concentrations between saliva and GCF, with higher levels observed in GCF, underscores the unique role of GCF in periodontal health. GCF, being in direct contact with periodontal tissues, likely reflects localized biochemical activity and immune responses more accurately than saliva. Studies have shown that GCF acts as a reservoir for antioxidants, including vitamin C, which helps neutralize ROS generated during inflammation [9, 29].

The elevated vitamin C levels in GCF compared to saliva may also be attributed to its role in collagen synthesis and tissue repair, as GCF is collected from periodontal pockets where active tissue degradation and repair occur. Vitamin C facilitates the synthesis of collagen necessary for the regeneration of periodontal ligaments and connective tissue, explaining its higher concentration in GCF. Additionally, the compartmentalization of body fluids might contribute to these differences, as GCF directly reflects the inflammatory and oxidative stress dynamics in periodontal tissues, while saliva provides a more generalized snapshot of oral health [30, 31].

Interestingly, inflammation-induced changes in local nutrient transport could also explain the higher vitamin C levels in GCF. Periodontal disease involves complex inflammatory processes, which may enhance vitamin C transport to GCF as part of the body's response to oxidative stress [32]. This aligns with findings in the literature that highlighted the role of inflammation in modulating antioxidant concentrations at the site of tissue damage [21, 33].

Although GCF exhibited higher median vitamin C concentrations compared to saliva, our analysis revealed that salivary vitamin C was a stronger independent predictor of periodontal health status. This finding aligns with the distinct physiological roles of these two fluids.

The differing trends observed between salivary and GCF vitamin C concentrations may be attributed to the distinct physiological origins and functions of these fluids. Saliva is produced by the salivary glands and represents a systemic reflection of antioxidant levels influenced by overall nutritional and metabolic status [19, 20]. Conversely, GCF is a localized inflammatory exudate derived from gingival tissues and capillaries at the site of periodontal inflammation. As such, it is more immediately responsive to local immune activity and oxidative stress [34]. This compartmentalization between systemic and local environments may explain why the two fluids do not always exhibit parallel trends. Additionally, variability in sample collection, such as the specific site selected for GCF extraction, may also contribute to fluctuations in measured concentrations. These differences highlight the need to interpret salivary and GCF biomarkers within their unique physiological contexts when assessing periodontal disease. Previous studies have emphasized saliva's value as a noninvasive diagnostic tool for systemic and oral conditions, highlighting its accessibility, integrative biochemical profile, and reproducibility [35, 36].

Despite the higher concentration of vitamin C in GCF compared to saliva, logistic regression analysis revealed that salivary vitamin C was more predictive of periodontal health status. The positive association between salivary vitamin C levels and periodontal health suggests that salivary vitamin C may serve as a more reflective and accessible biomarker of systemic antioxidant status [19]. In contrast, GCF vitamin C, although locally abundant, demonstrated minimal predictive contribution when adjusted for age and gender. This finding is consistent with literature indicating that saliva integrates systemic antioxidant dynamics more comprehensively than GCF, which primarily reflects localized inflammation. Prior studies have shown saliva's diagnostic strength in assessing oxidative stress, whereas GCF is more variable due to site-specific inflammation. Thus, despite its lower concentration, salivary vitamin C offers excellent predictive reliability and clinical utility, supporting its use as a diagnostic biomarker in periodontal assessment [32, 37, 38].

Variability in dietary vitamin C intake among participants may partially explain the observed differences in salivary and GCF vitamin C levels. While our study did not control for individual dietary habits, future research should integrate nutritional assessments or plasma vitamin C measurements to distinguish dietary influences from disease-related depletion.

A key limitation of this study is the relatively small sample size (*n* = 43), particularly when divided across four clinical severity groups. Although the sample size was determined using G^*⁣*^*∗*^^Power analysis for a medium effect size, the resulting statistical power (0.36) may be insufficient to detect subtle differences among the groups. This limitation may also contribute to borderline or nonsignificant findings in subgroup analyses, particularly for GCF vitamin C levels. Future research with larger, more diverse populations is recommended to validate and expand upon these findings. Second, as this is a cross-sectional study, it cannot establish causality between vitamin C levels and the progression of periodontal disease. Longitudinal studies are needed to determine whether vitamin C depletion contributes to disease development or merely reflects an ongoing inflammatory response. Third, the study did not account for potential confounding factors such as dietary vitamin C intake, systemic health conditions, and medication use, all of which may influence antioxidant levels. Future research should incorporate dietary assessments and biochemical markers of systemic vitamin C levels to better isolate the role of vitamin C in periodontal health. Additionally, the study focused solely on vitamin C as an oxidative stress marker without evaluating other key antioxidants or oxidative stress biomarkers, such as total antioxidant capacity (TAC) or malondialdehyde (MDA). A more comprehensive biochemical analysis, which includes multiple oxidative stress markers and inflammatory mediators, could provide deeper mechanistic insights. Lastly, saliva and GCF samples were collected at a single time point, which does not account for diurnal variations or dynamic fluctuations in vitamin C levels. Future studies employing repeated measurements or continuous monitoring could offer a more accurate understanding of how antioxidant levels fluctuate in relation to periodontal disease activity.

## 5. Conclusion

This study demonstrated a significant depletion of salivary vitamin C levels in periodontitis compared to healthy individuals, reflecting systemic oxidative stress. While no significant differences were observed in salivary vitamin C among periodontitis severity groups, GCF vitamin C levels were higher in both healthy and severe periodontitis, suggesting localized compensatory mechanisms. Additionally, the higher vitamin C levels in GCF compared to saliva emphasize the distinct roles of these fluids in periodontal health and disease.

## Figures and Tables

**Table 1:** Participant distribution based on 2017 classification and mapped severity.

Diagnosis (2017 classification)	Mapped to severity group	Count
Stage I grade A	Mild	5
Stage II grade A	Mild	4
Stage II grade B	Moderate	7
Stage III grade B	Moderate	5
Stage III grade C	Severe	2
Stage IV grade C	Severe	7
Healthy gingiva	Healthy	13
Total	—	43

**Table 2:** Shows the Kruskal–Wallis test to compare the medians of vitamin C levels in saliva across the groups.

Test	Statistic	*p*-Value	Severity	Median vitamin C (saliva)
Kruskal–Wallis	20.4599	0.000136	Healthy gingiva	4.7172
	—	—	Mild	0.8743
	—	—	Moderate	2.06
	—	—	Severe	3.4907

**Table 3:** The pairwise comparisons using Tukey's HSD.

Group 1	Group 2	Mean diff	*p*-adj	Lower	Upper	Reject
Healthy gingiva	Mild	−3.1	0	−4.6356	−1.5644	True
Healthy gingiva	Moderate	−2.6871	0.0001	−4.1048	−1.2695	True
Healthy gingiva	Severe	−1.6246	0.0346	−3.1602	−0.089	True
Mild	Moderate	0.4129	0.8927	−1.1487	1.9744	False
Mild	Severe	1.4754	0.0996	−0.194	3.1448	False
Moderate	Severe	1.0625	0.2769	−0.499	2.6241	False

**Table 4:** Shows the Kruskal–Wallis test to compare the medians of vitamin C levels in GCF across the groups.

Severity	Median vitamin C (GCF)	Kruskal–Wallis statistic	Kruskal–Wallis *p*-value
Healthy gingiva	4.9625	7.32	0.062
Mild	4.4719	7.32	0.062
Moderate	0.327	7.32	0.062
Severe	4.9625	7.32	0.062

**Table 5:** With the median levels of vitamin C in both GCF and saliva, along with the results of the Wilcoxon signed-rank test.

Fluid type	Median levels	Wilcoxon statistic	Wilcoxon *p*-value
Vitamin C in GCF	4.5537	284	0.021761
Vitamin C in saliva	3.1637	—	—

**Table 6:** Logistic regression analysis predicting periodontal health status based on vitamin C levels in gingival crevicular fluid (GCF) and saliva, adjusted for age and gender.

Predictor	Coefficient (*B*)	Odds ratio (*e^B^*)	Standard error	*p*-Value
Intercept	0.904	2.47	NA (perfect separation)	NA (perfect separation)
GCF vitamin C	−0.0018	0.998	NA (perfect separation)	NA (perfect separation)
Salivary vitamin C	0.0840	1.088	NA (perfect separation)	NA (perfect separation)
Age	−0.0118	0.988	NA (perfect separation)	NA (perfect separation)
Gender (male = 1)	−0.6412	0.527	NA (perfect separation)	NA (perfect separation)

*Note:* Although GCF showed higher median concentrations of vitamin C, salivary vitamin C demonstrated greater predictive strength (OR = 1.088). Perfect separation in the dataset prevented estimation of standard errors and *p*-values through conventional methods.

## Data Availability

The data that support the findings of this study are available upon request from the corresponding author. The data are not publicly available due to privacy or ethical restrictions.
